# Supplementary material for: Population-level deficit of homozygosity unveils CPSF3 as an intellectual disability syndrome gene
Source: Nat Commun. 2022 Feb 4;13:705. doi: 10.1038/s41467-022-28330-8 (PMC8817032; doi:10.1038/s41467-022-28330-8)
Supplement: Supplementary file 1 — Supplementary Information [file 41467_2022_28330_MOESM1_ESM.pdf]

Supplementary Information

Population-level deficit of homozygosity unveils CPSF3 as an intellectual disability syndrome gene

Arnadottir et al.

Supplementary Figure 1. Pedigree of patient A and patients C and D, homozygous carriers of *CPSF3* p.Glu468Gly.

Supplementary Figure 2. Pedigrees of the four genotypically confirmed homozygous carriers, and two suspected homozygous carriers, of *CPSF3* p.Gly468Glu (patients A-F).

Supplementary Figure 3. Pedigree of patients G and H, the two homozygous carriers of *CPSF3* p.Ile354Thr.

Supplementary Figure 4. CPSF3 mRNA expression in white blood cells of heterozygous carriers of *CPSF3* p.Gly468Glu (n=150), non-carriers (n=17,548), and in homozygous patient B (n=1).

Supplementary Figure 5. Western blot analysis of EBV transformed lymphocytes from Patient B, homozygous for *CPSF3* p.Gly468Glu, and five non-carriers.

Supplementary Table 1. Missense variants in known autosomal recessive (AR) disease genes[1], with a minor allele frequency (MAF) greater than 0.40% and a complete deficit of observed versus expected homozygous carriers in deCODE’s set of 153,054 chip-genotyped and imputed Icelanders.

Supplementary Table 2. Missense variants in *CPSF3* detected in homozygous state in patients with severe intellectual disability in Iceland and the US.

Supplementary Table 3. Full phenotypic information of two homozygous carriers of *CPSF3* p.Ile354Thr.

Supplementary Table 4. Distribution of genotypes in offspring of heterozygous carrier couples of the three homozygote deficit genotypes described in this study; *CPSF3* p.Gly468Glu, *GNE* p.Asp409Tyr, and *GLE1* p.Arg569His.

Supplementary References.

Supplementary Figures

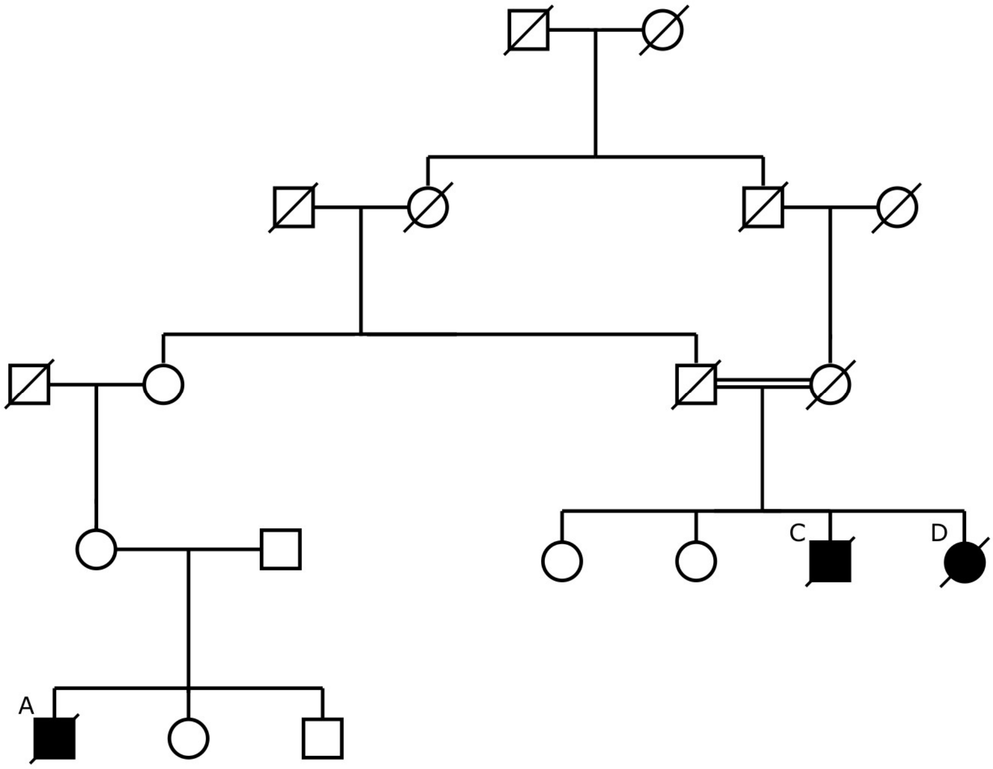

Supplementary Figure 1. Pedigree of patient A and patients C and D, homozygous carriers of *CPSF3* p.Glu468Gly. Squares represent males, circles represent females, a slashed symbol indicates a deceased individual, a double line indicate a consangineous union, and filled symbols represent affected individuals.

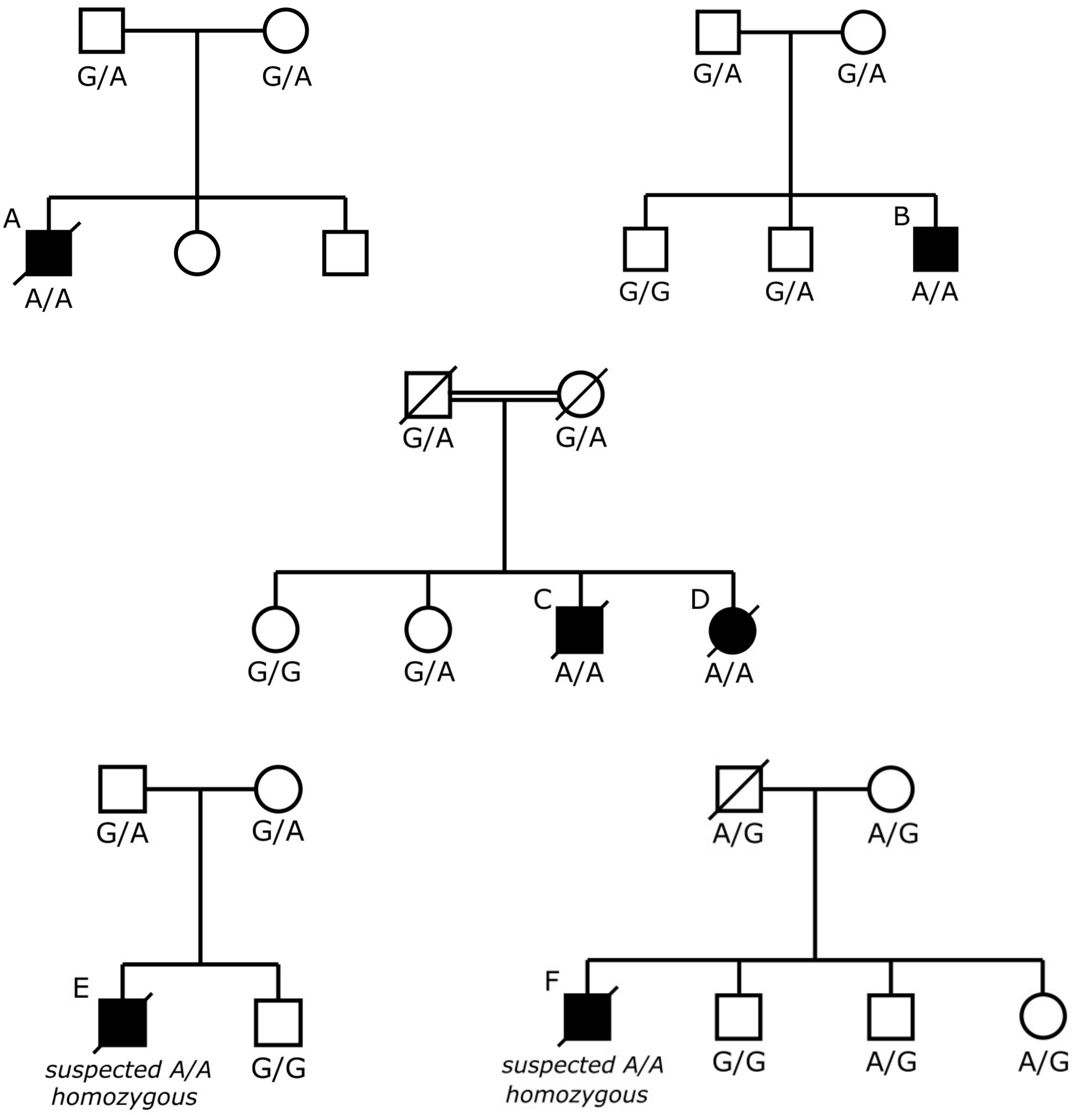

Supplementary Figure 2. Pedigrees of the four genotypically confirmed homozygous carriers, and two suspected homozygous carriers, of *CPSF3* p.Gly468Glu (patients A-F). The *CPSF3* p.Gly468Glu genotype status of all individuals that submitted a sample is indicated with G and A, G is the wild-type allele and A is the mutated allele. G/G therefore indicates non-carrier status, G/A indicates heterozygous carrier status, and A/A indicates homozygous carrier status. Squares represent males, circles represent females, a slashed symbol indicates a deceased individual, a double line indicate a consangineous union, and filled symbols represent affected individuals.

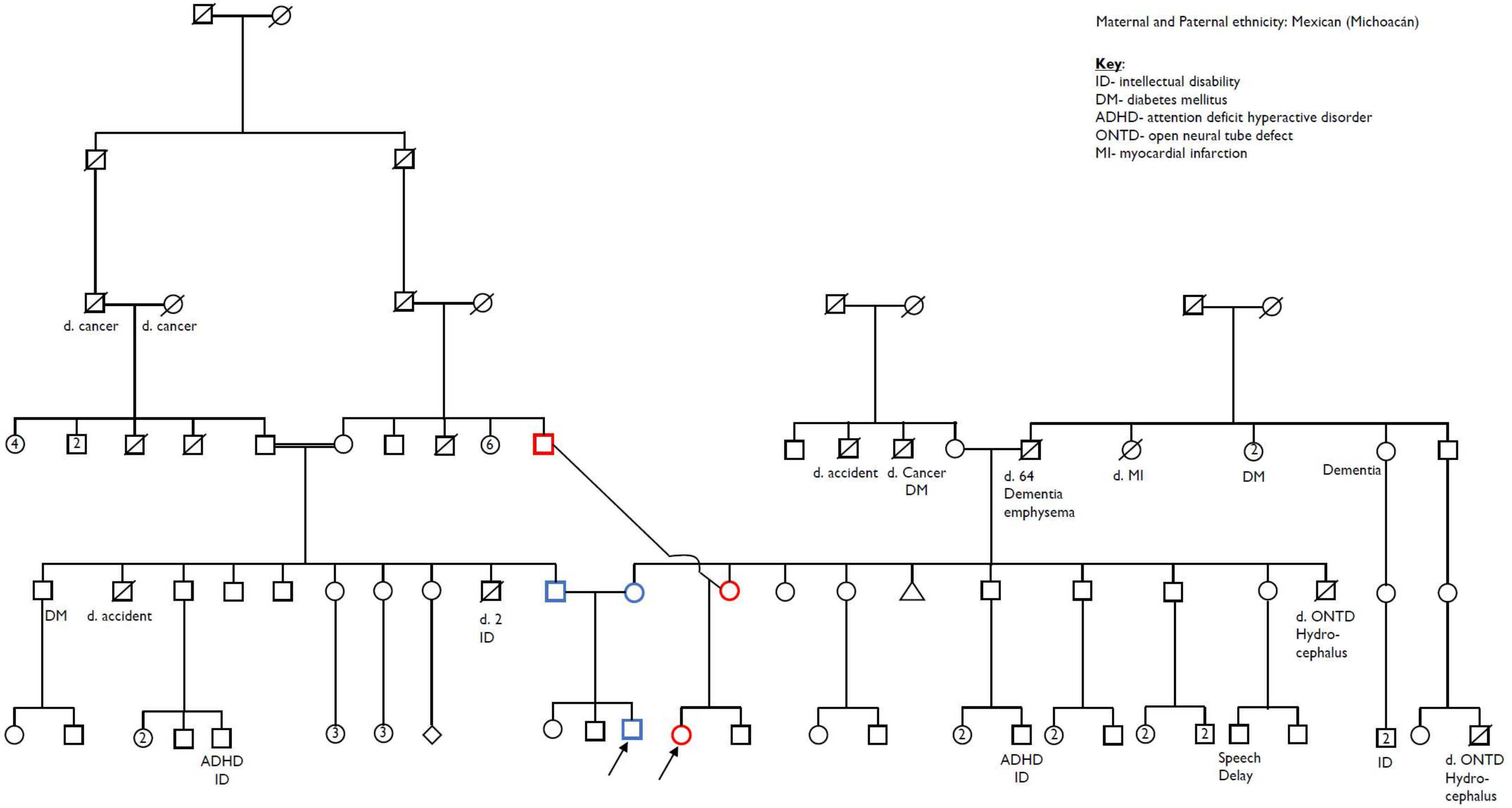

Supplementary Figure 3. Pedigree of patients G and H, the two homozygous carriers of *CPSF3* p.Ile354Thr. Squares represent males, circles represent females, a triangle represents a spontaneous abortion, and a diamond represents stillbirth. A slashed symbol indicates a deceased individual, and a double line indicates a consangineous union. Patients G and H are indicated with arrows, symbols for patient G and his parents are blue, symbols for patient H and parents are red.

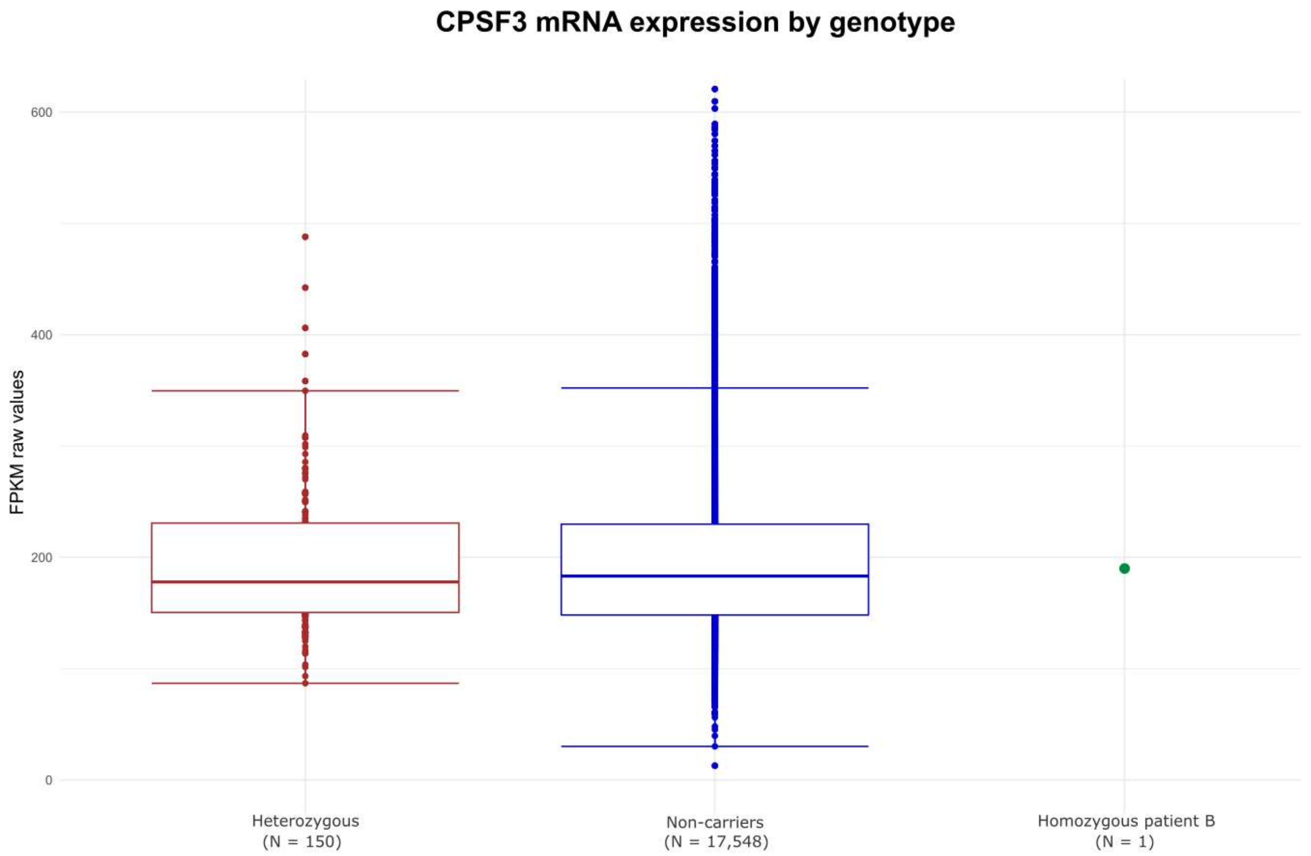

Supplementary Figure 4. CPSF3 mRNA expression in white blood cells of heterozygous carriers of *CPSF3* p.Gly468Glu (N=150), non-carriers (N=17,548), and in homozygous patient B (N=1). Expression values are fragments per kilo base of transcript per million mapped fragments (FPKM). There is no indication of reduced CPSF3 mRNA expression in heterozygous carriers (median FPKM = 177.93) compared to non-carriers (median FPKM = 183.20). We further assessed expression of *CPSF3* in white blood cells from one homozygous carrier, patient B, and observed no reduction in mRNA levels (mean FPKM over *CPSF3* is 191.80). The box plots show the median values (only a single value for patient B), and the bounds of the boxes correspond to the first (25%) and third (75%) quartiles. The whiskers mark the smallest and lowest values observed, limiting to 1.5 times the interquartile range. For ease of interpretation, the y axis is cut at 620 FPKM.

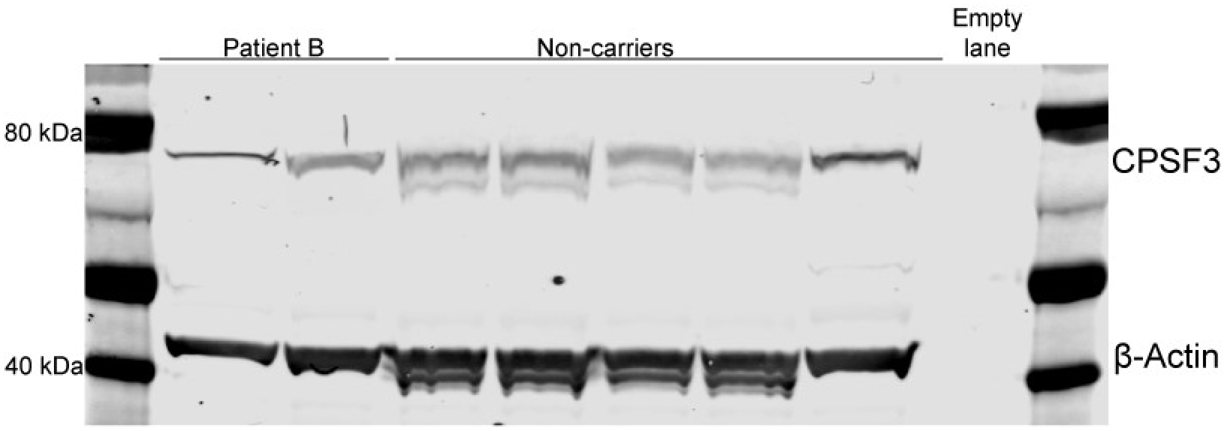

Supplementary Figure 5. Western blot analysis of EBV transformed lymphocytes from Patient B, homozygous for *CPSF3* p.Gly468Glu, and five non-carriers. The western blot confirms expression of the CPSF3 protein in a homozygous carrier of *CPSF3* p.Gly468Glu, as well as in non-carriers. Two lysates were analysed from Patient B. β-Actin was used as a loading control. Image is representative of two experiments and the scan is uncropped and unprocessed.

Supplementary Tables

Supplementary Table 1. Missense variants in known autosomal recessive (AR) disease genes[1], with a minor allele frequency (MAF) greater than 0.40% and a complete deficit of observed versus expected homozygous carriers in deCODE's set of 153,054 chip-genotyped and imputed Icelanders.

Position hg38	Ref	Alt	MAF (%)	Gene	HGVS	Observed homoz.	Expected homoz.	Known AR disease gene	Homozygous clinical case
chr9:128536414	G	A	0,82	*GLE1*	NP_001003722.1:p.Arg569His	0	10	yes	no
chr2:27242740	G	C	0,62	*CAD*	NP_004332.2:p.Val2115Leu	0	6	yes	no
chr11:71482875	A	G	0,61	*NADSYN1*	NP_060631.2:p.Thr393Ala	0	6	yes	no
chr9:36227397	C	A	0,60	*GNE*	NP_005467.1:p.Asp378Tyr	0	6	yes	yes
chr8:144360604	T	C	0,60	*SLC52A2*	NP_001350047.1:p.Leu339Pro	0	5	yes	yes
chr16:8811153	G	A	0,58	*PMM2*	NP_000294.1:p.Arg141His	0	5	yes	no
chr19:40714313	G	A	0,53	*COQ8B*	NP_079152.3:p.Arg63Trp	0	4	yes	no
chr3:33058265	T	G	0,52	*GLB1*	NP_000395.2: p.Glu186Ala	0	4	yes	yes
chr14:75005867	G	T	0,52	*EIF2B2*	NP_055054.1:p.Gly200Val	0	4	yes	no
chr21:46416552	C	T	0,51	*PCNT*	NP_006022.3:p.Arg2212Trp	0	4	yes	no
chr21:46265403	G	A	0,51	*MCM3AP*	NP_003897.2:p.Pro1051Leu	0	4	yes	no
chr16:66549968	G	A	0,51	*TK2*	NP_004605.4:p.Arg32Trp	0	4	yes	no
chr16:547925	T	C	0,51	*CAPN15*	NP_005623.1:p.Cys363Arg	0	4	yes	no
chr17:6703031	C	T	0,49	*SLC13A5*	NP_808218.1:p.Gly219Arg	0	4	yes	yes
chr17:80112072	G	A	0,49	*GAA*	NP_000143.2:p.Gly576Ser	0	4	yes	no
chr1:155962988	G	C	0,47	*ARHGEF2*	NP_001155855.1:p.Pro307Arg	0	3	yes	no
chr13:94465691	C	T	0,46	*DCT*	NP_001913.2:p.Asp269Asn	0	3	yes	no
chr21:42375787	C	T	0,45	*TMPRSS3*	NP_001243246.1:p.Ala425Thr	0	3	yes	no
chr12:15624339	C	T	0,43	*EPS8*	NP_004438.3:p.Ala705Thr	0	3	yes	no
chr2:27483350	C	T	0,43	*IFT172*	NP_056477.1:p.Gly170Asp	0	3	yes	no
chr1:200565127	T	A	0,43	*KIF14*	NP_055690.1:p.Glu1338Val	0	3	yes	no
chr1:114678495	C	A	0,43	*AMPD1*	NP_001166097.1:p.Met339Ile	0	3	yes	no
chr3:165046998	A	C	0,43	*SI*	NP_001032.2:p.Val577Gly	0	3	yes	no
chr3:12901269	G	A	0,42	*IQSEC1*	NP_001127854.1:p.Pro1020Leu	0	3	yes	no
chr10:16915832	T	C	0,42	*CUBN*	NP_001072.2:p.Asn2400Ser	0	3	yes	no
chr18:63351049	C	T	0,42	*KDSR*	NP_002026.1:p.Val150Met	0	3	yes	no
chr6:31743924	C	T	0,42	*MSH5*	NP_751898.1:p.Arg146Cys	0	3	yes	no
chr8:103931324	A	C	0,42	*RIMS2*	NP_001269810.1:p.Asp608Ala	0	3	yes	no
chr6:52028309	T	C	0,41	*PKHD1*	NP_619639.3:p.Tyr1136Cys	0	3	yes	no
chr7:117530975	G	A	0,41	*CFTR*	NP_000483.3:p.Arg117His	0	3	yes	no
chr5:83111112	T	C	0,41	*XRCC4*	NP_003392.1:p.Leu75Ser	0	3	yes	no
chr9:6644629	T	C	0,41	*GLDC*	NP_000161.2:p.Met107Val	0	3	yes	no
chr7:97020846	C	G	0,41	*DLX5*	NP_005212.1:p.Glu254Gln	0	3	yes	no
chr15:101178197	T	A	0,41	*CHSY1*	NP_055733.2:p.Ile534Leu	0	3	yes	no

Supplementary Table 2. Missense variants in *CPSF3* detected in homozygous state in patients with severe intellectual disability in Iceland and the US.

**Chromosomal position (hg38)**	chr2:9452920	chr2:9441942
**cDNA change[a]**	c.1403G>A	c.1061T>C
**Protein change**	p.Gly468Glu	p.Ile354Thr
**MAF Iceland (%)**	0.41	NA
**MAF Latino/Admixed American gnomAD (%)**	NA	$6 \cdot 10^{-3}$
**Observed / expected homozygous carriers**	0 / 3	NA
**GERP**	highly conserved (5.81)	highly conserved (5.78)
**SIFT**	deleterious	deleterious
**PolyPhen-2**	probably damaging	probably damaging

[a]RefSeq transcript NM_016207.3
MAF = Minor allele frequency.
GERP = Genomic Evolutionary Rate Profiling. Scores ranging from -12.36 to 6.18. In general, negative scores indicate non-conserved positions while positive scores scale with the level of constraint, the greater the score, the greater the level of evolutionary constraint. The percentile applies to all GERP scores for the coding sequence of the genome.
SIFT = Sorting intolerant from tolerant. SIFT and PolyPhen-2 are scores for the predicted effect of amino acid substitution on protein structure/function.

Supplementary Table 3. Full phenotypic information of two homozygous carriers of *CPSF3* p.Ile354Thr.

**Age at last assessment**	12 years	13 years
**Family history**	Double first cousin (mothers of patients are sisters, and fathers are nephew-uncle)	Double first cousin (mothers of patients are sisters, and fathers are nephew-uncle)
**Consanguinity**	Yes- paternal grandparents	No
**Affected siblings**	No	No
**Unaffected siblings**	Yes- no molecular testing at this time	Yes- no molecular testing at this time
**Neurologic / CNS**	Seizure-like activity and spasticity noted at 7 m. Clonazepam and baclofen started and continued ever since, with doses adjusted according to weight gain/spaticity. Most recent EEG at 12 y 8 m did not capture seizure activity. Peripheral motor neuropathy, docummented by initial NCV at 4y 5m with progression of severity docummented at 7 y 4 m.	Seizures, generalized tonic clonic, with abnormal EEG, diagnosed at 4 m. Treated with phenobarbital ever since. Breakthrough seizures when weaning off medication was attepted at 3 y 8 m. Most recent EEG at 10 y showed multifocal and generalized epileptiform discharges, consistent with Lennox Gastaut Syndrome. Peripheral motor neuropathy docummented by NCV at 8 y 5m.
**MRI findings**	**MRIs completed at 7 months, 4 years, 7 years of age**	**MRIs completed at 4 months, 2 years, 8 years of age**
	7 m: thinning of the corpus callosum, white matter volume loss; vermian hypoplasia within a normal sized posterior fossa.	4 m: no abnormalities identified
	4 y: progressive cerebral atrophy (white matter loss); T2 hyperintensity in periventricular white matter. Diffuse thinning of the corpus callosum. Mega cisterna magna, hypoplasia of the cerebellum (including the vermis).	2 y: Significant loss of white matter volume throughout both cerebral and cerebellar hemispheres with associated dilatation of the lateral ventricules. Patchy, predominantly peritrigonal T2 hyperintensity.Atrophy of the corpus callosum.
	7 y: Mild progression of the above - mentioned changes.	8 y: Redemonstration of diffuse white matter volume loss. Hypoplastic inferior vermis, and small cerebellar hemispheres. Asymmetric atrophy of the right mesial temporal lobe.
**Development**	Severe neurodevelopmental delay. Visual inattention noted before 6 m. No head control, never able to sit independently or crawl. No acquired language	Severe neurodevelopmental delay.Visual inattention noted before 4 m. No head control, never able to sit independently or crawl. No acquired language.
**Cognition**	Minimal interaction with surroundings. Responds music, voices, and is very sensitive to loud noises without startle response.	Minimal interaction with surroundings. Responds music, voices, and is very sensitive to loud noises without startle response.
**Gastrointestinal / feeding**	Feeding difficulties/ choking / ALTE at 2 m. Gastroesophageal reflux diagnosed at 15 m with no improvemt with medical treatment. Poor weight gain. G-tube placement and Nissen Fundoliplication at 2 y 8 m. Chronic constipation. Suffered transverse colon volvulus at 10 y 9 m, requiring right hemicolectomy with revision one month later due to bowel obstruction and adhesions. Currently is fully dependent on g-tube for feedings and medications.	Feeding and swallowing difficulties, poor weight gain, gastroesophageal reflux requiring medication. G-tube placed at 3 y 9m. Chronic constipation. G-tube dependent for feedings and medications.
**Immunological**	No known problems	No known problems

**Respiratory**	Chronic aspiration. Severe obstructive sleep apnea-hyponea syndrome, significant pharyngo-laryngo malacia. Requires frequent suctioning and respiratory treatments. Recently started on C-PAP.	Chronic aspiration. Requires frequent suctioning and respiratory treatments.
**Musculoskeletal**	Spasticity requiring treatment with Baclofen and Clonazepam since 7 m (dose adjusted according to weight gain and symptoms). Progressive contractures in hips requiring surgical release at 2 y 8 m. Bilateral hip dislocation identified at 7y 8m. Generalized osteopenia with secondary bone fractures.	Spasticity requiring treament with Baclofen since age 2 y. Progressive hip contractures and dislocations requiring multiple surgical interventions. Osteopenia.
**Renal / genitourinary**	-	Pyelonephritis at 7 y 4 m. No recurrence of urinary tract infections
**Endocrine**	Normal pubertal developmental	Started puberty, no menses yet
**Cardiac**	-	-
**Dysmorphic features**	Microcephalic, high palate, protruding teeth	Microcephalic, high palate, protruding teeth
**Prenatal manifestations**	Decreased fetal movements	Decreased fetal movements
**Other findings**	Visual inattention noted early in life. Bilateral primary optic atrophy with bilateral diffuse optic atrophy secondary to progressive neurodegenerative disease. Severe visual impairment. Bilateral alternating esotropia and vertical nystagmus.	Visual inattention noted at 2 m. Bilateral pallor of optic nerves, nystagmus, alternating esotropia. Cortical blindness.

Supplementary Table 4. Distribution of genotypes in offspring of heterozygous carrier couples of the three homozygote deficit genotypes described in this study; *CPSF3* p.Gly468Glu, *GNE* p.Asp409Tyr, and *GLE1* p.Arg569His. This data is extracted from the population set of 153,054 chip-genotyped and imputed Icelanders.

**Variant**	**Heterozygous carrier couples in population set (N)**	**Non-carrier offspring (% of genotyped offspring)**	**Heterozygous offspring (% of genotyped offspring)**	**Homozygous offspring**
CPSF3 p.Gly468Glu	3	1 (50%)	1 (50%)	0
GNE p.Asp409Tyr	10	5 (31%)	11 (69%)	0
GLE1 p.Arg569His	17	4 (24%)	13 (76%)	0

Supplementary References

1. McKusick, V. A. Mendelian Inheritance in Man and Its Online Version, OMIM. *Am. J. Hum. Genet.* **80**, 588–604 (2007).
