# Supplementary material for: Population-level deficit of homozygosity unveils CPSF3 as an intellectual disability syndrome gene
Source: Nat Commun. 2022 Feb 4;13:705. doi: 10.1038/s41467-022-28330-8 (PMC8817032; doi:10.1038/s41467-022-28330-8)
Supplement: Supplementary file 3 — Description of Additional Supplementary Files [file 41467_2022_28330_MOESM3_ESM.pdf]

Description of Additional Supplementary Files

File name: Supplementary Data 1.

Description: Missense variants with a minor allele frequency (MAF) greater than 0.40% and a complete deficit of observed versus expected homozygous carriers in deCODE‘s set of 153,054 chip-genotyped and imputed Icelanders.
