# Supplementary material for: Population-level deficit of homozygosity unveils CPSF3 as an intellectual disability syndrome gene
Source: Nat Commun. 2022 Feb 4;13:705. doi: 10.1038/s41467-022-28330-8 (PMC8817032; doi:10.1038/s41467-022-28330-8)
Supplement: Supplementary file 5 — Reporting Summary [file 41467_2022_28330_MOESM5_ESM.pdf]

nature portfolio

Corresponding author(s): Patrick Sulem, Kari Stefansson

Last updated by author(s): Jan 5, 2022

Reporting Summary

Nature Portfolio wishes to improve the reproducibility of the work that we publish. This form provides structure for consistency and transparency in reporting. For further information on Nature Portfolio policies, see our Editorial Policies and the Editorial Policy Checklist.

Statistics

For all statistical analyses, confirm that the following items are present in the figure legend, table legend, main text, or Methods section.

n/a	Confirmed	
☐	☒	The exact sample size (*n*) for each experimental group/condition, given as a discrete number and unit of measurement
☐	☒	A statement on whether measurements were taken from distinct samples or whether the same sample was measured repeatedly
☒	☐	The statistical test(s) used AND whether they are one- or two-sided<br>*Only common tests should be described solely by name; describe more complex techniques in the Methods section.*
☐	☒	A description of all covariates tested
☐	☒	A description of any assumptions or corrections, such as tests of normality and adjustment for multiple comparisons
☐	☒	A full description of the statistical parameters including central tendency (e.g. means) or other basic estimates (e.g. regression coefficient) AND variation (e.g. standard deviation) or associated estimates of uncertainty (e.g. confidence intervals)
☒	☐	For null hypothesis testing, the test statistic (e.g. *F*, *t*, *r*) with confidence intervals, effect sizes, degrees of freedom and *P* value noted<br>*Give P values as exact values whenever suitable.*
☒	☐	For Bayesian analysis, information on the choice of priors and Markov chain Monte Carlo settings
☒	☐	For hierarchical and complex designs, identification of the appropriate level for tests and full reporting of outcomes
☒	☐	Estimates of effect sizes (e.g. Cohen's *d*, Pearson's *r*), indicating how they were calculated

Our web collection on statistics for biologists contains articles on many of the points above.

Software and code

Policy information about availability of computer code

Data collection	We used the following publicly available software for data collection, as described in our Methods section:<br><br>BWA-MEM version 0.7.10, available at https://github.com/lh3/bwa<br>GenomeAnalysisTKLite version 2.3.9, available at https://github.com/broadgsa/gatk/<br>Picard tools version 1.117, available at https://broadinstitute.github.io/picard/<br>SAMtools version 1.3, available at http://samtools.github.io/<br>Variant Effect Predictor (release 100), available at https://github.com/Ensembl/ensembl-vep
Data analysis	We used the following publicly available software for data analysis, as described in our Methods section:<br><br>BWA 0.7.10 mem, https://github.com/lh3/bwa<br>GenomeAnalysisTKLite 2.3.9, https://github.com/broadgsa/gatk/<br>Picard tools 1.117, https://broadinstitute.github.io/picard/<br>SAMtools 1.3, http://samtools.github.io/<br>Variant Effect Predictor (release 100) https://github.com/Ensembl/ensembl-vep<br>BOLT-LMM (version 2.1) https://data.broadinstitute.org/alkesgroup/BOLT-LMM/downloads/<br>We used R (version 3.6.0) for data analysis and for creating plots.

For manuscripts utilizing custom algorithms or software that are central to the research but not yet described in published literature, software must be made available to editors and reviewers. We strongly encourage code deposition in a community repository (e.g. GitHub). See the Nature Portfolio guidelines for submitting code & software for further information.

Data

Policy information about availability of data

All manuscripts must include a data availability statement. This statement should provide the following information, where applicable:

- Accession codes, unique identifiers, or web links for publicly available datasets
- A description of any restrictions on data availability
- For clinical datasets or third party data, please ensure that the statement adheres to our policy

Our previously described Icelandic population whole-genome sequence data3 have been deposited at the European Variation Archive under accession PRJEB15197. The authors declare that the data supporting the findings of this study are available within the article, its supplementary files, and upon reasonable request (data containing sensitive information, such as miscarriage phenotypes etc. cannot be made publicly available).

Field-specific reporting

Please select the one below that is the best fit for your research. If you are not sure, read the appropriate sections before making your selection.

- [x] Life sciences
- [ ] Behavioural & social sciences
- [ ] Ecological, evolutionary & environmental sciences

For a reference copy of the document with all sections, see nature.com/documents/nr-reporting-summary-flat.pdf

Life sciences study design

All studies must disclose on these points even when the disclosure is negative.

Sample size	Samples sizes are clearly indicated in the Methods section of our manuscript. In brief, our study is based on a set of 153,054 chip-genotyped and imputed Icelanders, as well as 56,959 whole-genome sequenced Icelanders. Sample size was not predetermined for the purpose of this study, we used the available genotype dataset previously collected by deCODE genetics to calculate minor allele frequencies and expected homozygote frequencies, and from this we compiled our set of missense variants with a complete deficit of homozygous carriers in the genotype set.
Data exclusions	Individuals with either one or both parent of foreign ancestry were excluded from the study set, since the purpose of this study was to delineate founder variants that have reached elevated frequencies in the Icelandic population. We excluded all sequence variants with a minor allele frequency under 0.40%, since we wanted an expectation of at least 3 homozygous carriers; assuming Hardy-Weinberg equilibrium a minor allele frequency of 0.40% equals a homozygote frequency of 0.0016% which would correspond to 2.5 carriers in the genotype set of 153K Icelanders (rounded up this equals 3 homozygous carriers). We wanted to limit to high quality genotypes, and therefore only considered genotypes with imputation information over 0.9. For the purpose of this study, i.e. study on a deficit of homozygous missense variants, we excluded variants on the X chromosome. All of the data exclusions were predetermined based on our study design.
Replication	To be able to claim a novel disease gene, we sought to find genotypes from other population backgrounds that also resulted in the same phenotype as the Icelandic genotype. We succeeded in this by identifying two patients of Mexican descent who carried a homozygous variant in the same gene as the Icelandic patients (i.e. CPSF3). That variant is absent from the Icelandic population. Other forms of replication are not applicable to our study since our study is based on rare variants in Mendelian disease genes, present mainly by chance in a given patient sample set. We did not have other patient sample sets available for testing.
Randomization	No randomization was used, since our study is centered on monogenic causes of disease with little or no environmental effects.
Blinding	For the purpose of estimating the effect of the variants with a complete deficit of homozygous carriers in the population set on miscarriage, the health professional performing medical records lookup was blinded to the genotypic status of individuals.

Reporting for specific materials, systems and methods

We require information from authors about some types of materials, experimental systems and methods used in many studies. Here, indicate whether each material, system or method listed is relevant to your study. If you are not sure if a list item applies to your research, read the appropriate section before selecting a response.

Materials & experimental systems

n/a	Involved in the study	
[ ]	[x]	Antibodies
[x]	[ ]	Eukaryotic cell lines
[x]	[ ]	Palaeontology and archaeology
[x]	[ ]	Animals and other organisms
[ ]	[x]	Human research participants
[x]	[ ]	Clinical data
[x]	[ ]	Dual use research of concern

Methods

n/a	Involved in the study	
[x]	[ ]	ChIP-seq
[x]	[ ]	Flow cytometry
[x]	[ ]	MRI-based neuroimaging

Antibodies

Antibodies used	CPSF3 antibody (Abcam, #ab72295), beta Actin antibody (Abcam, #ab6276), mouse antibody (LI-COR, 926-32212) and rabbit antibody (LI-COR, #926-68073).
Validation	CPSF3 antibody (Abcam, #ab72295) validation according to manufacturers website (https://www.abcam.com/cpsf73-antibody-ab72295.html): Reacts with mouse and human species, suitable for WB, IP, IHC-P, validated on positive controls by manufacturer WB: HeLa, HEK293T, NIH 3T3. IHC-P: human breast carcinoma, mouse teratoma tissues. IP: HeLa cells.<br><br>beta Actin antibody (Abcam, #ab6276) validation according to manufacurers website (https://www.abcam.com/beta-actin-antibody-ac-15-ab6276.html): Knockout validated, reacts with Mouse, Rat, Cow, Dog, Human, African green monkey, Chinese hamster. Validated on positive controls by manufacturer: ICC/IF: SV40LT-SMC cells WB: HAP1, HeLa, Jurkat, A431, HEK-293, COS-7, NIH/3T3, PC-12, Rat2, CHO, MDBK and MDCK cell lysates.

Human research participants

Policy information about studies involving human research participants

Population characteristics	Our study is based on A) a WGS set of 56,959 Icelanders (controls) B) a chip-genotype set of 153K Icelanders, and C) a clinical WGS set of 2,501 individuals (patients and their close relatives).<br>A) & B) The WGS and chip-genotype sets consist of individuals participating in various disease projects at deCODE genetics, both cases and controls. The genotype set mainly consists of healthy adult individuals, 88% are alive at the time of this study, mean age of living individuals is 58.45 years, and 54% are female. Currently, the Icelandic population consists of around 340K individuals, and so this set represents close to half of the population and as such is not enriched for any specific diseases or traits. The WGS set is a subset of the chip-genotype set.<br>C) The clinical WGS set consists of 764 patients of (mainly) assumed monogenic disorders and their close family members, all in all 2,501 individuals. Diagnoses include, but are not limited to, epilepsy, intellectual disability, autism, deafness, blindness, immune dysfunction, and cardiac anomalies. The clinical WGS set was recruited in the period 2014-2021, the mean age of the patients is 26,4 years, and 46% are female. Samples from patients and family members, submitted to WGS, are from whole-blood, buccal tissue, or purified DNA. WGS and genotyping of the set of 2,501 was performed as described in our Methods section.
Recruitment	Individuals included in the Icelandic population sets (WGS and/or chip-genotyped) provided samples as volunteers in various disease projects, either as cases or controls. Samples were collected at various locations as part of a nationwide participation in genetic research led by deCODE genetics. The chip-genotype set represents close to half the population of Iceland, with no bias in recruitment (apart from individuals being older than 18 years at the time of sample acquisition).<br><br>The set of clinically WGS individuals by means of clinical diagnostics, mainly through the University Hospital of Iceland.
Ethics oversight	As stated in our Methods section:<br>All participating individuals who donated blood or buccal tissue samples, or their guardians, provided written informed consent. All sample identifiers were encrypted in accordance with the regulations of the Icelandic Data Protection Authority. Personal identities of the participants and biological samples were encrypted by a third-party system approved and monitored by the Icelandic Data Protection Authority. The study was approved by the Data Protection Authority (ref. 2013030423/ÞS/--, with amendments) and the National Bioethics Committee (ref. VSN-19-023, VSNb2019010015/03.01), that also reviewed and approved the protocol, methodology and all documents presented to the participants.

Note that full information on the approval of the study protocol must also be provided in the manuscript.
